# Editorial: A year in review: discussions in human and medical genomics

**DOI:** 10.3389/fgene.2025.1618183

**Published:** 2025-05-20

**Authors:** Maxim B. Freidin, Jared C. Roach

**Affiliations:** ^1^ Department of Twin Research and Genetic Epidemiology, King’s College London, London, United Kingdom; ^2^ Institute for Systems Biology, Seattle, WA, United States

**Keywords:** genetic diversity, genomics of breast cancer, tele-genetics, genetic counselling, precision medicine

This Research Topic compiles manuscripts inspired by five highly viewed papers published in Human and Medical Genomics section of Frontiers in Genetics in 2022 (Ashenhurst et al., 2022; Gee et al., 2021; Jimenez-Kaufmann et al., 2021; Schmidlen et al., 2022; Van der Merwe et al., 2022). These five papers applied different methodology and consider different aspects of basic and applied research in fast advancing areas of human genetics, including the development of a polygenic risk score to improve risk prediction for type 2 diabetes (Ashenhurst et al., 2022), getting insight into the pathophysiology of sickle cell anaemia in children via genome-wide transcriptome analysis (Gee et al., 2021), expanding and improving an imputation panel for Latin Americans via increased inclusion of Native American populations (Jimenez-Kaufmann et al., 2021), discovering the reasons for low uptake in cascade genetic testing for monogenic diseases and potential mitigation strategies (Schmidlen et al., 2022), and estimating the prevalence of germline driver mutations in *BRCA* genes and developing an effective diagnostic test tailored for African populations using national healthcare facilities (Van der Merwe et al., 2022).

Four out of five of these highly viewed papers focused on non-European populations, a greatly welcomed trend in contemporary genetic research owing to the fact that to date the vast majority of research in human genetics has been carried out in European (Caucasian) populations. Analysing non-European populations in medical genetics is crucial for advancing equitable healthcare. Including diverse ancestries enhances the accuracy of genetic predictions, supports the discovery of novel, population-specific variants, and ensures that advances in precision medicine benefit all communities, reducing global health disparities. These analyses also aid development of more inclusive reference genomes and diagnostic tools, ultimately leading to more effective, personalized treatments across ethnicities and improving global health outcomes through better-informed medical decisions.

The four papers in this Research Topic follow this trend. Wu et al. leveraged publicly available genomic databases to analyse mutations in ethnically divergent populations in the *CTNS* gene (OMIM 606272) responsible for the rare disease cystinosis. They identified allele-frequency discrepancies for some variants in different populations and found that the variant c.124G>A (ClinVar ID 4447) would be classified as non-pathogenic due to its high frequency in an African population despite that fact that this variant is known to be causing an atypical form of the disease with incomplete penetrance and variable expressivity. The higher frequency of the c.124G>A variant in the African population raises concerns about racial disparities and inequitable healthcare, potentially leading to underdiagnosis of atypical cystinosis. If symptoms go unrecognized due to bias or limited access to care, the variant may be misclassified as benign despite clinical relevance. Authors postulated that access to healthcare should be considered a confounding factor in variant classification, and a more inclusive diagnostic approach across diverse populations is essential for accurate assessment of pathogenicity and equitable care.

Chu et al. reviewed the prospects of genetic counselling via the Hong Kong Genome Project focusing on tele-genetics, the approach in genetic counselling based on videoconferencing. The need for this approach in Asia is dictated by the lack of specialists in genetic counselling in the region. Authors review evidence that tele-genetic counselling is an effective way to provide this important service and is also cost effective. Despite being a valuable approach, the development of tele-genetics in Asia remains slow; also, patients from Asian countries demonstrate lower preference for telemedicine compared to Western countries. Factors that affect the receptivity of tele-genetics in Asia include age, socioeconomic background, attitude, health, and technological literacy. Specifically concerning Hong-Kong: its small size, dense population, and well-developed commute infrastructures slow down the acceptance of telemedicine. Authors argue that the Hong Kong Genome Project will serve as a platform to catalyse the development of genomic medicine and genetic counselling with tele-genetics playing more remarkable role in healthcare.

Two papers published in this Research Topic are devoted to genetics and genomics of breast cancer in Sub-Saharan African populations. The mini review by Ndiaye et al. discusses the mutation spectrum in breast cancer driver genes in black African women in contrast with Caucasian populations. While *BRCA1* and *BRCA2* were the major drivers both in African and Caucasian women, their contribution was greater among the native black African women and black women from the African diaspora compared with Caucasian women. The relative impact of other known breast cancer driver genes, such as *TP53* and *PALPB2*, also varied. Based on these findings, authors urge to expand molecular investigation of breast cancer mutation profiles in large cohorts of patients from Sub-Saharan populations to facilitate personalised therapy and improved patient care. Another mini review by Oosthuizen et al. is focused on practical implementation of genetic testing for breast cancer mutations in South Africa. It discusses the implementation of cost-effective, population-directed point-of-care genetic testing, particularly for BRCA1/2 founder mutations, to enhance accessibility in primary healthcare settings. The authors also explore the use of multigene panels and exome sequencing to broaden the scope of genetic screening, despite limitations in infrastructure and resources. They emphasize the importance of haplotype analysis to improve risk assessment and personalized treatment strategies. They highlight that a cost-effective, population-directed portable genotyping assay accompanied by tele-counselling could enable rural screening, increasing access to genetic testing and improving primary care in developing healthcare systems.

**FIGURE 1:**
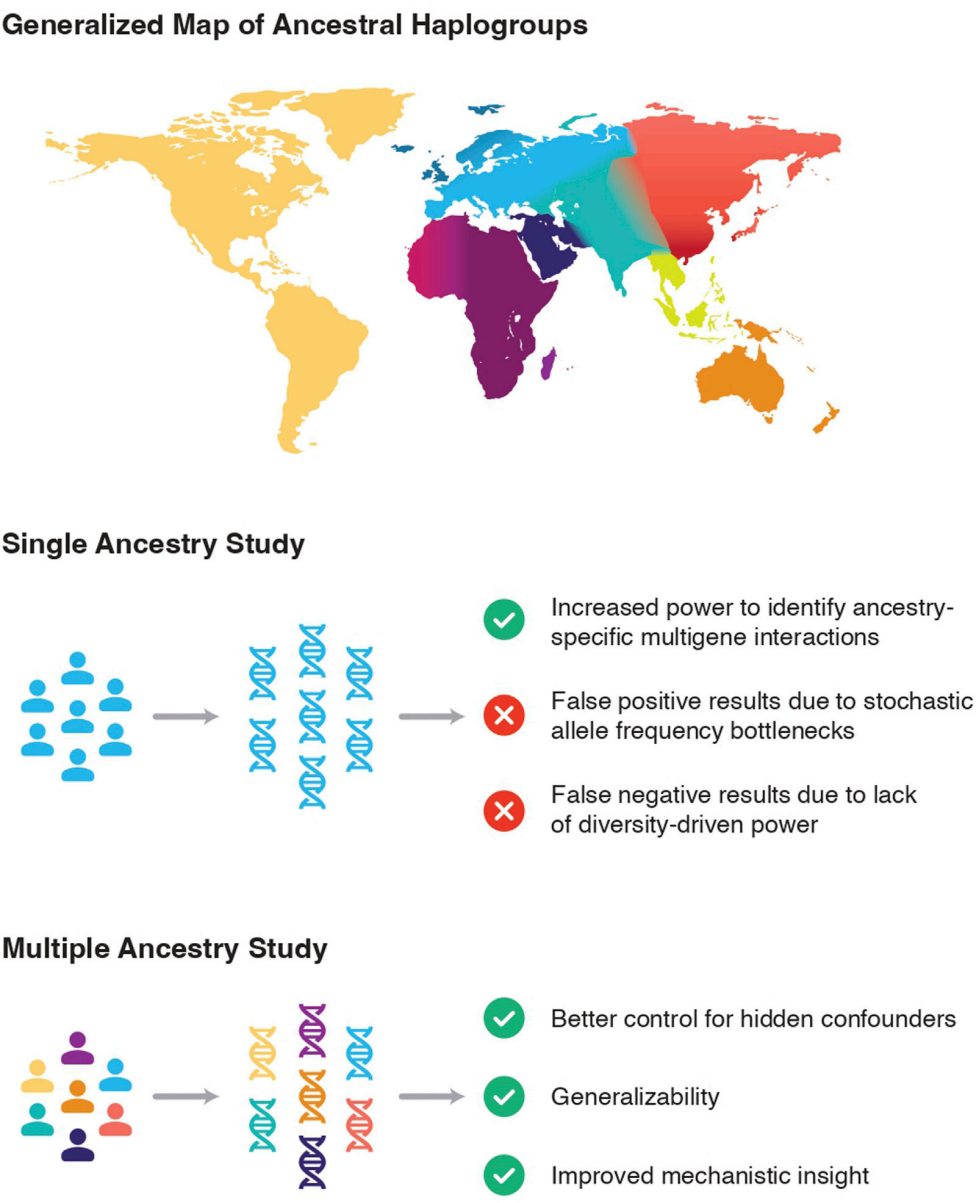
The impact of increasing diversity in medical genetic studies.

The featured papers in this Research Topic address a historic imbalance in genetic research and aim to improve equity in precision medicine. The included studies emphasize the importance of genetic diversity in understanding disease risk, improving diagnostic accuracy, and tailoring treatments. The perspectives emphasize the urgent need for inclusive genomic research to close healthcare gaps and ensure that medical advancements benefit all populations globally (Figure 1).

Shifting our perspective from this past year to the future of human and medical genomics, the papers published in this Research Topic underscore the need to broadly and openly share genomic data following findable, accessible, interoperable, reusable (FAIR) principles. Genetic data is of little use in gated silos. Great insights remain to be gained by collaboration between human scientists and artificial intelligence leveraging vast medical and omics datasets (Fecho et al., 2022). These analyses will increasingly focus on complex multigenic diseases and on drivers of human health and wellness requiring genomewide perspectives (Roach and Freidin, 2023).
